# The missing middle: Private laboratories in Cameroon’s quality management landscape

**DOI:** 10.4102/ajlm.v15i1.3407

**Published:** 2026-10-13

**Authors:** Ebai Besong Ebot

**Affiliations:** 1 Independent Researcher, Yaoundé, Cameroon

Laboratory Quality Management Systems (LQMS) have transformed clinical diagnostic reliability across sub-Saharan Africa over the past two decades, with documented implementation experiences from Tanzania, Ethiopia, Nigeria, Benin, the Democratic Republic of Congo, Morocco, and Cameroon. Yet this growing evidence base has a persistent and largely unaddressed blind spot: it is drawn almost entirely from public and donor-supported facilities, and it contains almost no information about the private diagnostic laboratory sector, which accounts for approximately one-third of health facilities in Cameroon.^1^ Drawing on operational experience directing laboratory quality systems across a 30-facility private diagnostic network in Cameroon, this paper argues that private laboratories represent a ‘missing middle’ in Africa’s laboratory quality discourse, and outlines why closing this evidence gap matters for patient safety and regional laboratory policy.

## Africa’s laboratory quality journey: Remarkable progress, uneven evidence

The past two decades have witnessed remarkable progress in strengthening LQMS across Africa. Driven by growing recognition of the central role of laboratory medicine in clinical care, disease surveillance, outbreak preparedness, and public health decision-making, numerous countries have implemented structured quality improvement programmes aimed at enhancing diagnostic reliability and pursuing international accreditation. Regional initiatives such as the Strengthening Laboratory Management Toward Accreditation programme, the Stepwise Laboratory Quality Improvement Process Towards Accreditation, and broader adoption of ISO 15189 have transformed laboratory quality management from a largely technical exercise into a strategic component of health-system strengthening. By 2024, 674 laboratories across 28 African countries had achieved Stepwise Laboratory Quality Improvement Process Towards Accreditation certification, illustrating the scale of this progress.^2^

Published implementation studies consistently show that structured interventions can produce substantial gains in laboratory performance, even in resource-constrained settings. Improvements have been linked to strong institutional leadership, structured mentorship, routine internal auditing, corrective and preventive action programmes, continuous monitoring of quality indicators, and systematic process redesign. Evidence from Tanzania remains among the earliest and most influential demonstrations of successful LQMS implementation, illustrating how sustained mentorship facilitated progress towards ISO 15189 accreditation at a national reference facility.^3^ Similar experiences from Ethiopia showed that health-centre laboratories in resource-constrained settings could achieve measurable quality gains through structured implementation and continuous performance monitoring.^4^ Comparable findings have been reported from Nigeria,^5^ Benin,^6^ and the Democratic Republic of Congo,^7^ alongside a Clinical and Laboratory Standards Institute-led mentorship programme spanning laboratories in Africa and Central Asia,^8^ and Morocco.^9^ In Cameroon, a widely cited case study documented how the Strengthening Laboratory Management Toward Accreditation programme transformed the Bamenda Regional Hospital Laboratory between 2009 and 2013.^10^

Despite this encouraging evidence, important limitations remain. Most implementation studies have been conducted in public hospitals, national reference laboratories, or donor-supported institutions. Many describe single facilities or small groups of laboratories, limiting understanding of how quality systems perform across larger, more heterogeneous networks. Relatively few studies examine long-term sustainability once external mentorship or donor support concludes.

One particularly notable feature of the existing evidence base is the limited representation of private diagnostic laboratories. Although private laboratories provide an increasingly important share of diagnostic services across African health systems, comparatively few published studies examine how quality management systems are implemented, financed, or sustained within commercially operated laboratory environments. This imbalance does not diminish the substantial achievements documented within public-sector laboratory strengthening programmes; rather, it suggests that the published evidence currently reflects only part of Africa’s laboratory ecosystem – one in which Cameroon’s own documented contribution, though real, remains confined to the public hospital sector.

The observations presented in this paper are informed by a narrative reading of the published literature and the author’s professional experience in laboratory quality management, rather than by a systematic review. The intention is to stimulate discussion of an underexplored area of laboratory quality implementation in Africa, not to resolve it.

## The missing middle: Why private laboratories are different, and why that matters

Private diagnostic laboratories operate under fundamentally different incentives than the public and donor-supported facilities that dominate the existing literature. A donor-funded reference laboratory’s quality investment is typically driven by accreditation targets tied to grant conditions; a private laboratory’s investment must instead compete against short-term margin pressure, out-of-pocket payment models and, in many markets, limited regulatory enforcement.

This creates a genuinely different set of questions. Does a quality intervention’s measured impact, such as error rate or turnaround time, differ when the underlying incentive is commercial rather than donor- or government-driven? Can test-utilisation stewardship, meaning the reduction of unnecessary or duplicate testing, be implemented where test volume is tied directly to revenue, without a structural conflict of interest? And what happens to quality gains once external mentorship or grant-driven urgency lapses, in a facility that must fund any continued investment from patient revenue alone?

In practice, many private Cameroonian laboratories already operate informal, partial quality-management practices: ad hoc internal audits driven by an individual quality champion, informal tracking of corrective actions without a documented corrective and preventive action system and, in some cases, participation in external quality assessment on an individual rather than programme-wide basis. What typically distinguishes these efforts from accreditation-ready quality management is not the absence of quality awareness, but the absence of documentation and continuity: standard operating procedures that persist independent of any one staff member, an internal audit cycle that runs on a fixed schedule rather than opportunistically, and a corrective and preventive action system that creates an auditable record. Bridging this gap is less a matter of introducing quality concepts from scratch than of formalising practices that already exist in nascent form – a distinction with direct implications for how support programmes for this sector should be designed.

This experience draws on a 30-facility private diagnostic network in Cameroon, comprising approximately 3000 tests processed monthly and a quality team of 20 laboratory professionals. There, the application of Lean Six Sigma Define–Measure–Analyse–Improve–Control methodology was associated with a 15% reduction in error rates and a 29% improvement in average turnaround time. These figures are offered as illustrative field experience rather than formal, peer-reviewed research data, intended to motivate the research agenda rather than as generalisable findings.

## Why Cameroon, and why now?

Cameroon’s healthcare landscape includes a substantial private diagnostic laboratory sector serving patients across urban centres and secondary cities, alongside public hospital laboratories and donor-supported reference facilities. As noted, a published case study documents LQMS implementation in Cameroon’s public hospital sector.^10^ But no peer-reviewed study, in Cameroon or, to this author’s knowledge, anywhere in Central Africa, examines LQMS implementation in a private diagnostic laboratory network as its central research question, nor draws cross-institutional comparisons among private laboratories with differing ownership and governance models. This is not to say the private sector is entirely undocumented: commercial materials from at least one private Cameroonian laboratory describe active quality-management efforts towards future ISO 15189 accreditation,^11^ and a peer-reviewed evaluation of Cameroon’s Strengthening Laboratory Management Toward Accreditation training model included one private laboratory, added in 2010 alongside four public facilities, in a cohort whose central research question concerned training methodology rather than private-sector dynamics.^12^ Neither constitutes a study examining private-sector laboratory quality management as its central research question, which remains the gap this paper identifies.

This omission matters: as the Africa Centres for Disease Control and Prevention expand laboratory strengthening initiatives, policy recommendations built solely on public-sector evidence risk misalignment with how a large share of Central African patients actually access diagnostic services.^1^

## Toward a research and policy agenda

This paper does not resolve these questions; it names them clearly enough to be studied rigorously. A research agenda should include:

- Comparative, multi-site evaluation of quality intervention outcomes across private laboratory networks with differing ownership and governance structures in a single Central African country, using a shared outcome framework, such as turnaround time, pre-analytical error rate, and proficiency testing performance, to allow cross-site comparison;
- Development of a test-utilisation stewardship model suited to commercially financed diagnostic settings, designed so that recommendations do not conflict with a facility’s revenue model, drawing on lessons from public–private partnership models documented elsewhere in African diagnostic services^13,14,15^; and
- Longitudinal tracking of quality-gain sustainability once external mentorship or grant support ends, since private facilities, unlike most donor-supported sites in the literature, must fund continued investment from patient revenue alone.

## Conclusion

Africa’s laboratory quality movement has made real, well-documented progress over the past two decades. But its evidence base has a shape: institutionally concentrated in the public and donor-funded sector, with private diagnostic laboratories comparatively unstudied across the continent, including in Cameroon. Closing this gap is not merely an academic exercise. It matters for the safety of patients served by an expanding private diagnostic sector, and for the calibration of regional laboratory strengthening policy going forward.
